# Effects of DL and L-Methionine on Growth Rate, Feather Growth, and Hematological Parameters of Tetra-SL Layers from 1–28 Days of Age

**DOI:** 10.3390/ani12151928

**Published:** 2022-07-28

**Authors:** James Kachungwa Lugata, János Oláh, Xénia Erika Ozsváth, Renáta Knop, Eszter Angyal, Csaba Szabó

**Affiliations:** 1 Department of Animal Nutrition and Physiology, Faculty of Agriculture and Food Sciences and Environmental Management, University of Debrecen, Böszörményi Street 138, 4032 Debrecen, Hungary; jkachungwa@agr.unideb.hu; 2 Doctoral School of Animal Science, Faculty of Agriculture and Food Sciences and Environmental Management, University of Debrecen, Böszörményi Street 138, 4032 Debrecen, Hungary; ozsvath.xenia@agr.unideb.hu (X.E.O.); angyal.eszter@agr.unideb.hu (E.A.); 3 Institutes for Agricultural Research and Educational Farm, University of Debrecen, Böszörményi Street 138, 4032 Debrecen, Hungary; olahja@agr.unideb.hu; 4 Department of Animal Husbandry, Faculty of Agriculture and Food Science and Environmental Management, University of Debrecen, Böszörményi Street 138, 4032 Debrecen, Hungary; dr.knop.renata@agr.unideb.hu

**Keywords:** DL-methionine, L-methionine, blood hematology, Tetra-SL

## Abstract

**Simple Summary:**

Methionine is the first limiting amino acid and plays an important role in the growth and health status of chicks. Animal health status was easily detected by examining the hematological parameters. This study aimed to investigate the influence of methionine sources (L-methionine and DL-Methionine) at different supplementation levels (10% deficit, 100% and 10% surplus of the recommended) on growth performance and health status of chicks. The results of the feeding trial found no differences in the chicks’ growth performance and feather growth on neither methionine sources nor methionine levels. However, most of the hematology parameters were affected by the methionine sources and/or methionine levels, as well as their interaction being significant in some cases. Therefore, this trial showed that varying methionine by ±10% of the requirements does not adversely affect growth performance and feather growth.

**Abstract:**

The study was carried out to determine whether sources or levels of methionine (Met) affect the health status of Tetra-SL (TSL) chicks by examining growth performance, feather growth, and hematological parameters. A total of ninety-six (96) day-old (44.2 ± 0.18 g lw) TSL chicks were randomly allotted to six treatment groups (three levels for each DL and L-Met source) with four replicates of four chicks each. Chicks were fed ad libitum diets supplemented with 90, 100, and 110% of methionine requirements for four weeks after hatch. The parameters examined are bodyweight (BW), average daily gain (ADG), feather length (FL), and hematological parameters, including: red blood cell (RBC) count, hemoglobin (Hb) concentration in the blood, hematocrit (Ht; %), number of white blood cells (WBC), platelet count, mean corpuscular volume of red blood cells (MCV), mean corpuscular hemoglobin (MCH), mean corpuscular hemoglobin concentration (MCHC), lymphocyte (LYM), mid-range (MID), and granulocyte (GRAN). There was no significant effect of Met sources and levels on BW, ADG, and FL of TSL chicks for the first four weeks of rearing. The RBC, Hb, Ht, WBC, LYM, MID, and GRAN values of TSL chicks were statistically influenced by dietary Met sources and Met levels (*p* < 0.05). Among the treatment groups, the number of white blood cells (WBC) on 90% DL–Met was the lowest. WBC, RBC, Hb, and Ht were higher from chicks that received 100% DL-Met than all other levels, regardless of the Met sources. The MCV, MCH, and MCHC values were not affected by either Met source or levels or their interactions. Met source and level interactively affected the Ht, WBC, LYM %, and GRAN values of TSL chicks (*p* < 0.05). The platelet number was affected by Met source only (*p* < 0.05) with chicks receiving L-Met source having more than twofold higher platelet values than DL-Met source. In conclusion, varying Met levels by ±10% of the requirement does not adversely affect the growth performance, feather growth, and hematological parameters of TSL chicks reared for up to four weeks of age. DL-Met increased the body weight and improved the white blood cells, red blood cells, and hematocrit at 28 days after hatch. DL-Met showed similar biological efficacy as L-Met for body weight and feather growth but not for the hematological parameters.

## 1. Introduction

Amino acids (aa) are one of the critical components in poultry diets, especially sulfur aa, which are essential for growth and feather development of poultry [1,2]. Methionine (Met) is the first limiting amino acid in plant-based poultry diets with potential roles, including; protein synthesis for feather growth, improving performance, antioxidant status, cellular metabolism, and immunological function [3,4,5,6,7,8]. Feathers play a vital role in birds by preventing skin from abrasions and infections as well as by providing insulation and hence minimizing the maintenance energy needed. Several studies have reported that methionine and cysteine are involved in synthesizing feather keratin and are crucial for feather growth [9,10]. The most commonly used sources of Met in poultry diets are DL-methionine (DL-Met) and its methionine hydroxyl analog-free acid (MHA) [11], which has been available for over 40 years [12]. However, recently, L-methionine (L-Met) produced by fermentation has been made available as a feed additive for poultry nutrition [13,14]. The latter is the only Met source readily available for immediate cell metabolism, while DL-Met and MHA must first be converted to L-Met to be used by the cell [15,16]. Growth performance has been used as an indicator of the chickens’ health status [17]. Dietary Met sources and levels determine the young birds’ growth performance and the production performance in later stages [18]. However, many experiments which have been carried out to assess the efficacy of MHA to DL-Met in poultry and swine have inconclusive results [19,20,21]. A few experiments have observed that L-Met is 1.4-times more bio-efficient than DL-Met for growth performance of starter ducks and development of broiler chicks [22,23,24]. Nevertheless, studies on the efficacy of DL-Met relative to L-Met involving high-performance layers at the early growth stage are relatively limited.

In addition, hematological parameters have been referred to for basic diagnostics of health status in mammals but have rarely been used in birds [25,26]. The hematological parameters reveal the physiological status of the animal concerning its internal and external environment. This includes the nutrient supply of the animal, which can be characterized by normal values of the hematological parameters, while abnormal values may indicate poor nutrition [25]. To our knowledge, limited studies have been conducted to examine the efficacy of L-Met relative to DL-Met in TSL growing chicks as well as their influence on physiological health status. Therefore, this experiment aimed to determine the effects of dietary DL-Met and L-Met supplementation at different levels on body weight (BW), average daily gain (ADG), feather growth, and hematological parameters of Tetra-SL LL chicks reared up to four-weeks of age.

## 2. Materials and Methods

This trial was carried out at the Institutes for Agricultural Research and Educational Farm of the University of Debrecen. The experimental protocol and procedures were reviewed and approved by the Animal Care Committee of the University of Debrecen (6/2021/DEMÁB).

### 2.1. Experimental Animals and Dietary Treatments

The Tetra-SL LL (TSL) breeder eggs were obtained from Bábolna Tetra Ltd. (Bábolna, Hungary) and incubated at standard temperature and humidity (37.8 °C and 60% Rh, PLM 3600, PL Maschine KFT Budapest, Hungary). After hatching, a total of 96 one-day-old chicks (44.2 ± 0.18 g lw) were weighed and distributed to six treatments, with four replicates having four chicks (125 cm^2^/bird) per pen. The experimental design was a complete randomized block with a 2 × 3 factorial arrangement (2 Met sources × 3 Met levels). The chicks had similar initial body weight for each treatment and pen. Feed and water were provided ad libitum. The pens were bedded with wood shavings. Extra heating in the pens was provided by infrared lamps (optima plus II 175 W), and the temperature in the pens was maintained according to the breeder’s recommendation [27]. The chicks were provided ad libitum with a standard mash diet that was formulated according to the manual guidelines of TSL, except for the Met levels. The diets were formulated to include 90, 100, and 110% of either DL (MetAmino, feed grade 99%, Evonik Degussa GmbH, Wesseling, Germany) or L-Met (L-Met 100, feed grade 99%, CJ Europe GmbH, Schwalbach/Taunus, Germany) of the nutrient requirements of the breeders (Table 1) [27]. 

### 2.2. Measurements

All chicks in each pen were included in the measurement of the length of the fourth primary feather and body weight weekly for four weeks [9]. The length of the feather was measured in millimeters using a vernier caliper with 0.01 mm accuracy. The average feed intake and feed conversion ratio were not calculated in this study due to the immeasurable amount of feed waste and the fact that the experiment utilized layer genotype. No mortalities were observed for the entire experimental period.

### 2.3. Collection of Blood and Hematological Analysis

At the end of the trial (28th day of life), 2 chicks from each pen (a total of 8 birds per treatment group) were randomly selected. Blood was collected from the cutaneous ulnar vein (also known as the brachial wing vein) into EDTA-coated tubes following the procedures of Kelly and Alworth [28]. The blood samples were immediately placed on ice and transferred to the laboratory. Before analysis, blood samples were allowed to come to room temperature and were gently mixed, then 20 µL of blood was pipetted and added to 1 mL of dilution buffer. Hematological parameters, such as the number of red blood cells (RBC), hemoglobin (Hb) concentration in the blood, hematocrit (Ht; %), the number of white blood cells (WBC), and platelet count were determined. In addition, the following mean corpuscular volume of red blood cells (MCV), mean corpuscular hemoglobin (MCH), mean corpuscular hemoglobin concentration (MCHC), lymphocyte (as percentage and count) (LYM), mid-range (MID), and granulocyte (GRAN) were obtained. The hematological parameters were analyzed by using an automated hematology analyzer (URIT-3000 Vet Plus, Orvostechnika Ltd., Budapest, Hungary). Reading was performed in triplicate and the average of the runs was taken. 

### 2.4. Statistical Analysis

Before conducting statistical analyses, data were checked for outliers by using the median absolute deviation method (MAD) [29] and normality tests were performed. The experimental unit for ADG was the pen (*n* = 4), while for BW, FL (*n* = 16), and hematological parameters (*n* = 8 per treatment), the individual birds were considered an experimental unit. SAS OnDemand For Academics (https://www.sas.com/it_it/software/on-demand-for-academics.html (last accessed on 21 July 2022) GLM procedure two–way analysis of variance was used to analyze all data. Tukey’s multiple comparison test was performed when analyses of variance indicated a significant difference. The *p*-value of *p*< 0.05 was considered statistically significant.

## 3. Results

### 3.1. Growth Rate

Except for the interaction effect in week 4 (Table 2), neither Met source nor Met levels influenced the bodyweight of the chicks throughout the experiment. Moreover, the dietary Met source and Met level did not have a significant influence on the ADG except for the fourth week of age, where the interaction was noted, and Met levels tended to affect ADG. In week 4, the highest ADG value (14.6 g/day) was obtained in the DL-Met group with a supplementation level of 110%, which increased by 7.6% from the DL-Met supplementation at 100% of recommendation (Table 2).

### 3.2. Feather Development

Met source and level had no effect on feather development as a main effect; however, their interaction was significant or tended to be significant at all data collection days (Table 3). Generally, the 110% L-Met group showed the shortest feather length at each measurement as compared to other groups. On the contrary, the DL-Met group with a 110% inclusion level revealed better feather development with the highest feather length (86.72 mm) in week 4.

### 3.3. Hematological Parameters

The number of erythrocytes (RBC) in the blood of chicks was significantly influenced by Met source (*p* = 0.004) (Table 4). The Met concentrations also influenced the number of RBCs (*p* = 0.016). However, there was no significant interaction between Met source and levels and the number of RBC. There was a significant difference in the Hb concentration between the Met sources (*p* = 0.001) and among the Met levels (*p* = 0.013). As with RBC, there was no significant interaction effect of Met source and levels on the Hb concentration of chicks’ blood at four-weeks of age.

The Ht value was significantly affected by both Met source (*p* = 0.005) and levels (*p* = 0.007) with a demonstration of the interaction of Met source and Met levels (*p* = 0.024). However, there were no significant differences in MCV, MCH, and MCHC values exerted by either dietary Met sources, levels, or their interaction. The dietary Met source significantly affected the platelet count in blood liters (*p* = 0.001) with the higher values observed in L-Met as compared to DL-Met. No significant effects of Met levels or interactions of Met sources and Met levels were revealed. The highest value of platelet count was obtained in the L-Met group with 110% inclusion (10.44 × 10^9^/L), while the lowest was in the DL-Met group at the same dose (2.33 × 10^9^/L) (Table 4).

The WBC count in the blood liter was affected by both Met sources (*p* = 0.003), Met levels (*p* = 0.009), and their interaction (*p* = 0.001). The percentage of lymphocytes was also significantly influenced by both dietary Met source and dietary Met levels with their interaction, the same as for WBC count. Unlike the percentage of lymphocytes, the number of LYM cells was only affected by Met sources (*p* = 0.044) and levels (*p* = 0.023) with no interaction effect observed. While the MID percentage was not influenced by the source or the levels, the MID number was significantly affected by both factors but not by their interactions (Table 4).

Similarly, the percentage of GRAN % was statistically affected by both Met source (*p* = 0.014) and levels (*p* = 0.012) with no interaction effect. However, the GRAN cell count was significantly affected by Met-source (*p* = 0.005) and Met levels (*p* = 0.001, as well as having a significant interaction (*p* = 0.002). The highest levels of dietary inclusion in the L-Met group resulted in the lowest number and percentage (26.18 × 10^9^/L and 37.63%, respectively) of GRAN as compared to other dietary inclusion levels in both sources (Table 4).

## 4. Discussion

Growth performance has been used to determine the bioavailability (digestion, absorption, and utilization) of amino acids, particularly methionine, which is the first limiting amino acid in maize- and soybean-meal-based poultry diets [23,30]. Our results indicate that young chicks of TSL have similar utilization of DL-Met and L-Met for the first four weeks of life on body weight and feather growth, as well as body weight gain. These results concur with several findings which have indicated that chicks can utilize DL-Met with the same efficacy as L-Met in weight gain [31]. Moreover, the study by Jankowski et al. [32] on turkeys showed no influence of dietary Met sources on the growth performance of the birds. On the contrary, the findings by Shen et al. [23] showed that L-Met was utilized better than DL-Met for intestinal development and growth of young broilers. They indicated that chicks required 138 units of DL-Met to obtain the overall ADG that was produced by 100 units of L-Met. In addition, Park et al. [33] reported better performance of L-Met than DL-Met in young turkeys from 0 to 28 days of age with a relative bioavailability of 160% for weight gain. Another study by Shen et al. [30] showed that young nursery pigs from days 26 to 46 days of age achieved 144% better ADG when fed L-Met than DL-Met. However, the results of our study at the fourth week of age show that the chicks who received DL-Met with an inclusion level of 110% had higher BW and ADG in the treatment groups. In comparison to the control group in the DL-Met source, the highest inclusion level improved the BW of the chicks by approximately 23%, while in the L-Met source, growth was depressed by 7% by the highest level of inclusion when compared to the control group. Generally, variation in the dietary Met levels by ± 10% of the strain’s requirement does not affect the BW and ADG of TSL chicks from 1 to 28 days of age.

Moreover, another objective of this trial was to determine the effect of DL-Met and L-Met supplementation at different levels on the feather development (length) in TSL chicks. The results showed that neither Met source nor Met levels affected the feather length development in the chicks from the first week to the third week of rearing. However, in the fourth week, the interaction of source and level was observed. These findings are in agreement with the results reported by Chen et al. [10] where no effect of DL-Met and L-Met supplementation was observed on the featherweights of broiler chicks. Similarly, Zhao et al. [34] and Zeng et al. [9] reported that DL-Met and DL-HMTBA showed the same efficiency for feather growth in Cherry Valley ducks and Pekin ducks, respectively. Our findings indicate that, as with BW, dietary DL and L-Met sources have similar efficacy on the feather development of TSL chicks until 28 days of age.

The other main objective of this study was to investigate the effects of DL-Met and L-Met on the hematological parameters of TSL chicks. Met source and levels both had a significant impact on blood parameters, both separately and in combination. No similar study was found on the effect of Met sources and levels on the TSL genotype. Hematological tests are commonly used in mammals to assess the health status of the animal but are not common in birds due to a lack of generally accepted reference values [25].

The assessment of RBC numbers is an essential element in hematological diagnostics which detects the malfunctioning of the circulatory system [35]. The study revealed that high levels of methionine might cause changes in complete blood count [36,37]. In this study, the RBC parameters were within the reference range regardless of the Met source and dietary Met levels. The chicks receiving a high dose of either source methionine had a slightly lower RBC number (Table 4) which was statistically significantly different from the other groups. However, the RBC counts of TSL chicks in DL-Met ranged from 2.82–3.42 × 10^12^/L and for L-Met ranged from 2.49–3.02 × 10^12^/L, which are within the recommended upper limit range of 2.5–3.5 × 10^6^/µL for healthy chickens reported by Mamba et al. [38]. Furthermore, similar findings were reported by Simaraks et al. [39] and Ding et al. [40], who discovered that RBC counts in Thai indigenous chickens and Chinese Haidong chickens ranged from 2.0 to 3.0 × 10^6^/L, respectively. Furthermore, Tombarkiewicz et al. [36] found that the number of RBC in broilers varied with age, ranging from 1.70–2.37 × 10^6^/L to 1.80–1.96 × 10^6^/L to 2.72–2.91 × 10^6^/L on the 1st, 7th, and 35th day of life, respectively. Higher Hb levels confirm the observed effect of Met source and levels on RBC numbers. However, the Hb values of TSL chicks amongst the six treatments ranged from 9.63 to 13.80 g/dL and are within the reference range of 7.0 to 13.0 g/dl of healthy chickens [41]. The Ht estimates the percentage of RBC in the body of the individual, also referred to as packed cell volume. The Ht value was lower in the 90% and 110% DL or L-Met doses than in the 100% dose. Generally, the Ht value ranged from 30.10 to 44.53%, which was affected by both Met source and level [42].

The elevated RBC parameters that include MCV, MCH, and MCHC are believed to be due to blood cell maturation disorders, such as megaloblastic and hemolytic anemia. However, in this study, MCV and MCH were not influenced by either Met source or Met level. This corroborates findings from the study by Chen et al. [43] where the hematological parameters in adult birds were not affected by either Met alone or in combination with zinc. The MCH values in this study ranged from 38.42 to 39.73 pg, which are within the normal values of 33–47 pg for healthy chickens [38]. In blood samples, MCH, the mean volume of hemoglobin per RBC, is mostly used to indicate the extent of anemia [44]; while MCHC, which is used to estimate the concentration of hemoglobin in RBC, was within the range of 30.39 to 31.43 g/dL, which falls into the accepted range [38,42,45]. The platelet number in this study ranged from 2.33 to 10.44 × 10^9^/L and was influenced by dietary Met sources.

Several studies have shown that the WBC count in adult chickens ranges from 12–30 × 10^3^/µL [35] or 9–32 × 10^3^ /µL [42], but in this experiment, 28-day-old chicks were used and the WBC ranged from 69.73–92.34 × 10^9^/L. This result correlates with the findings of Tombarkiewicz et al. [36] in young chickens with a WBC count ranging from 17–107 × 10^3^ /µL in response to the injection of methionine in the eggs. The influence of methionine at the 100% inclusion level had the highest WBC number than either the 90 or 110% level in both Met sources, indicating the immunological status of the chicks. The higher value of WBC suggests the ability of the chicks to fight or resist diseases, which reflects adequate methionine [38,46]. Furthermore, the study by Adeyemo et al. [46] indicated the influence of Met on hematological parameters, including WBC, in the first 4-weeks of rearing broilers. On the contrary, Zhang et al. [47] showed that Met supplementation of broiler diets did not affect WBC differential count. The percentage of lymphocytes in a total WBC count was noted to be higher (46.3%) in Met deficient diets (90%) for DL-Met source, while for L-Met it was observed to be high (50.24%) in Met excess diets (110%). This could imply that high doses of L-Met improved the chicks’ ability to fight diseases and the recovery process [38]. Granulocytes (GRAN), which play an important role in pathogen defense, were influenced by Met source and levels. DL-Met resulted in a higher number and percentage of GRAN than L-Met, while the highest Met level of inclusion had the lowest percentage and number of GRAN compared to other levels. This means that the ability of the Tetra-SL chicks to fight infection was altered by dietary Met source and levels. Chicks that received L-Met and a higher level of inclusion were immunologically stable.

## 5. Conclusions

In conclusion, supplementing the pre-starter diet with 90% of the recommended Met level could maintain growth performance and support feather growth the same as 100% and 110% Met levels of either DL or L Met from 1 to 28 days of age for TSL chick. Generally, DL-Met improved the hematological parameters of TSL chicks reared to 28 days of age compared to L-Met supplementation. This research suggests that DL-Met and L-Met have similar effects on early TSL chicks’ development and feather growth, implying that they may have the same biological efficacy for average daily gain and body weight with different utilization for hematological parameter responses. The anticipated higher bioavailability of L-Met could not be justified.

## Figures and Tables

**Table 1:** Composition (%) and calculated nutrient concentration (g/kg) in experimental diets formulated with different methionine inclusion levels of the recommendation.

Component		DL-Met			L-Met		
		90%	100%	110%	90%	100%	110%
Corn		59.79	59.84	59.88	59.79	59.84	59.88
Soybean meal, 46%		27.99	27.92	27.86	27.99	27.92	27.86
Fishmeal 65%		5	5	5	5	5	5
Sunflower oil		3.64	3.62	3.6	3.64	3.62	3.6
Limestone		1.13	1.13	1.13	1.13	1.13	1.13
MCP		1.61	1.61	1.61	1.61	1.61	1.61
Salt		0.3	0.3	0.3	0.3	0.3	0.3
L-Lys		-	-	-	-	-	-
DL-Met		0.04	0.08	0.12	-	-	-
L-Met		-	-	-	0.04	0.08	0.12
Vit. and mi. premix ^a^		0.5	0.5	0.5	0.5	0.5	0.5
Total		100	100	100	100	100	100
	Requirement	Calculated nutrient content					
AMEn, MJ/kg	12.35	12.35	12.35	12.35	12.35	12.35	12.35
CP	20.0	20.0	20.0	20.0	20.0	20.0	20.0
sidLys	1.00	1.008	1.007	1.005	1.008	1.007	1.005
sidMet	0.40	0.36	0.40	0.44	0.36	0.40	0.44
sidThr	0.63	0.65	0.65	0.65	0.65	0.65	0.65
sidTrp	0.20	0.42	0.42	0.42	0.42	0.42	0.42
Ca	1.00	1.00	1.00	1.00	1.00	1.00	1.00
Available P	0.48	0.48	0.48	0.48	0.48	0.48	0.48
Na	0.17	0.17	0.17	0.17	0.17	0.17	0.17
Met/Lys	0.40	0.357	0.397	0.438	0.357	0.397	0.438
Thr/Lys	0.63	0.647	0.647	0.647	0.647	0.647	0.647
Trp/Lys	0.20	0.413	0.414	0.414	0.413	0.414	0.414

^a^ 1 kg premix provided: 3,000,000 NE vitamin A, 600,000 NE vitamin D_3_, 14,700 mg/kg vitamin E, 600 mg vitamin K_3_, 450 mg vitamin B_1_, 150 mg vitamin B_2_, 3600 mg Ca-d-Pantothetane, 1200 mg vitamin B_6_, 7 mg vitamin B_12_, 33 mg biotin, 7507 mg niacin, 180 mg folic acid, 84,000 mg choline chloride, 19,800 mg Zn, 2880 mg Cu, 14,418 mg Fe, 19,800 mg Mn, 270 mg I, 63 mg Se, 18 mg Co.

**Table 2:** Effects of different Met sources and levels on growth rate in Tetra-SL layer chicks. (*n* = 4/treatment for ADG and *n* = 16/treatment for BW).

Treatment		BW on Day 1, g	BW on Day 7, g	ADG, g/day	BW on Day 14, g	ADG, g/day	BW on Day 21, g	ADG, g/day	BW on Day 28, g	ADG, g/day	ADG, g/day
Effect of Met source				Week 1		Week 2		Week 3		Week 4	Overall
DL-Met		42.39	78.22	5.11	124.05	6.50	205.01	11.57	271.05	10.34	8.39
L-Met		42.38	76.77	5.04	117.86	6.03	197.37	11.41	265.85	9.48	8.02
Effect of Met level											
90%		42.36	77.58	5.02	121.88	6.75	203.69	11.69	260.83	8.38	7.99
100%		42.40	78.27	5.21	120.59	5.97	198.27	10.67	263.15	9.50	8.04
110%		42.40	76.71	4.98	120.55	6.16	202.04	12.15	281.30	11.74	8.59
Effect of interaction source x level											
DL-Met	90%	42.35	77.86	5.04	125.63	6.69	205.59	11.42	250.46 ^a^	8.22 ^a^	7.88
	100%	42.41	77.68	5.04	120.76	6.16	197.85	11.02	255.49 ^a,b^	8.23 ^a^	7.61
	110%	42.41	79.06	5.24	125.78	6.67	211.61	12.26	306.87 ^b^	14.56 ^b^	9.68
L-Met	90%	42.37	77.36	5.00	118.13	6.84	201.67	12.04	270.5 ^a,b^	8.52 ^a,b^	7.90
	100%	42.37	79.00	5.39	120.40	5.71	198.78	10.20	271.33 ^a,b^	10.76 ^a,b^	8.47
	110%	42.38	74.03	4.72	115.19	5.65	191.84	12.00	255.72 ^a,b^	8.91 ^a,b^	7.49
RMSE		2.96	7.81	0.58	18.84	0.87	34.85	2.05	51.07	2.62	1.27
*p*-value	Met sources	0.984	0.403	0.783	0.120	0.260	0.304	0.867	0.638	0.404	0.504
	Met levels	0.998	0.676	0.692	0.948	0.217	0.839	0.388	0.240	0.064	0.607
	Interaction	0.999	0.281	0.351	0.558	0.442	0.485	0.813	0.013	0.017	0.067
	Model	1.000	0.563	0.691	0.580	0.318	0.704	0.792	0.040	0.022	0.214

BW—body weight, ADG—average daily gain, RMSE—root mean square error. ^a,b^ Means in a column with similar superscript do not differ (*p* > 0.05).

**Table 3:** Effects of different dietary Met sources and levels of supplementation on the fourth primary feather length of Tetra-SL layer chicks up to four-weeks of age. (*n* = 16/treatment).

Effect of Met Source		Feather length on Day 7 (mm)	Feather Length on Day 14 (mm)	Feather Length on Day 21 (mm)	Feather Length on Day 28 (mm)
DL-Met		38.58	57.50	73.80	84.94
L-Met		36.67	55.99	72.74	84.24
Effect of Met level					
90%		36.32	56.46	72.98	84.98
100%		39.71	57.88	73.80	83.87
110%		36.85	55.92	73.08	84.96
Effect of interaction source x level					
DL-Met	90%	35.37	55.76	73.37	84.96
	100%	40.38	58.13	73.09	83.14
	110%	39.99	58.62	74.96	86.72
L-Met	90%	37.26	57.20	72.54	85.01
	100%	39.04	57.62	74.51	84.59
	110%	33.71	53.23	71.07	83.09
RMSE		7.07	5.24	3.83	4.34
*p*-Value	Source	0.190	0.170	0.171	0.431
	Level	0.125	0.313	0.625	0.522
	Source x Level	0.072	0.033	0.027	0.063
	GLM-model	0.053	0.052	0.080	0.187

RMSE—root mean square error.

**Table 4:** Effect of different dietary Met source and levels of supplementation during the first four weeks of life on the hematological parameters of Tetra-SL chicks at 28-d of age. (*n* = 8/treatment).

		RBC (10^12^/L)	Hb (g/dL)	Ht (%)	MCV (fL)	MCH (pg)	MCHC g/dL	Platelet (10^9^/L)	WBC (10^9^/L)	LYM (%)	MID (%)	GRAN (%)	LYM (10^9^ cells/L)	MID (10^9^ cells/L)	GRAN (10^9^ cells/L)
Effect of Met source															
Dl Met		2.99 ^b^	11.91 ^b^	38.42 ^b^	127.66	39.5	30.76	2.70 ^a^	84.49 ^b^	45.09 ^a^	13.04	41.77 ^b^	38.35 ^b^	11.06 ^b^	36.15 ^b^
L Met		2.77 ^a^	10.99 ^a^	35.67 ^a^	127.70	39.11	30.84	8.00 ^b^	77.39 ^a^	47.45 ^b^	12.97	40.05 ^a^	36.06 ^a^	9.92 ^a^	31.85 ^a^
Effect of Met level															
90%		2.94 ^a,b^	11.4 ^a,b^	36.56 ^a,b^	126.79	38.62	30.70	5.54	78.82 ^a^	46.50 ^b^	13.17	40.47 ^a,b^	35.41 ^a^	10.02 ^a^	32.52 ^a^
100%		2.97 ^b^	11.96^b^	38.77 ^b^	129.21	39.69	30.68	3.86	86.67 ^b^	44.57 ^a^	12.85	42.36 ^b^	39.21 ^b^	11.27 ^b^	38.01 ^b^
110%		2.76 ^a^	11.05 ^a^	35.62 ^a^	126.55	39.56	31.01	6.76	78.94 ^a^	47.61 ^b^	13.02	39.97 ^a^	37.04 ^a,b^	10.22 ^a,b^	31.69 ^a^
Effect of interaction source x level															
DL-Met	90%	2.97	11.49	36.14 ^a,b^	126.53	38.85	30.69	2.52	76.54 ^a,b^	46.30 ^a^	13.18	40.59	35.32	10.05	31.17 ^a^^,b^
	100%	3.09	12.51	40.17 ^b^	128.03	39.73	30.98	3.22	92.34 ^c^	43.56 ^a^	12.97	42.90	40.13	11.75	40.15 ^c^
	110%	2.91	11.68	37.99 ^b^	128.14	39.77	30.60	2.33	86.83 ^b,c^	45.36 ^a^	13.00	41.65	39.59	11.22	36.42 ^b,c^
L-Met	90%	2.91	11.32	36.81 ^a,b^	126.95	38.42	30.71	9.06	81.11^a,b,c^	46.68 ^a^	13.15	40.36	35.48	9.99	33.87 ^b,c^
	100%	2.85	11.31	37.14 ^b^	130.58	39.64	30.39	4.50	82.13 ^b,c^	45.57 ^a^	12.71	41.72	38.29	10.70	35.52 ^b,c^
	110%	2.54	10.15	32.31 ^a^	125.28	39.31	31.43	10.44	69.73 ^a^	50.24 ^b^	13.04	37.63	34.50	9.06	26.18 ^a^
RMSE		0.21	0.77	2.55	3.68	1.05	0.73	4.09	6.69	1.95	0.39	2.13	3.27	1.36	4.08
*p*-Value	Met-Source	0.004	0.001	0.005	0.981	0.365	0.715	0.001	0.003	0.001	0.512	0.014	0.044	0.004	0.005
	Met-Levels	0.016	0.013	0.007	0.170	0.049	0.470	0.310	0.009	0.001	0.124	0.012	0.023	0.015	0.001
	Source x Level	0.240	0.086	0.024	0.259	0.893	0.073	0.124	0.001	0.020	0.586	0.090	0.146	0.065	0.002
	GLM -model	0.006	0.001	0.001	0.282	0.182	0.225	0.004	0.0002	0.0001	0.357	0.006	0.016	0.002	0.0001

RBC—red blood cells, Hb—hemoglobin, Ht—hematocrit, MCV—mean corpuscular volume of red blood cells, MCH—mean corpuscular hemoglobin, MCHC—mean corpuscular hemoglobin concentration, WBC—white blood cells, LYM—lymphocyte, MID—mid-range, GRAN—granulocyte, Met—methionine. ^a,b,c^ Means in a column with similar superscript do not differ (*p* > 0.05).

## Data Availability

The data presented in this study are available on request from the corresponding author.
